# Scaling Laws in Polysaccharide Rheology: Comparative Analysis of Water and Ionic Liquid Systems

**DOI:** 10.1021/acs.biomac.4c01125

**Published:** 2024-09-16

**Authors:** Roshan Akdar Mohamed Yunus, Daniele Parisi

**Affiliations:** Department of Chemical Engineering, Engineering and Technology Institute Groningen, University of Groningen, Nijenborgh 3, 9747 AG Groningen, The Netherlands

## Abstract

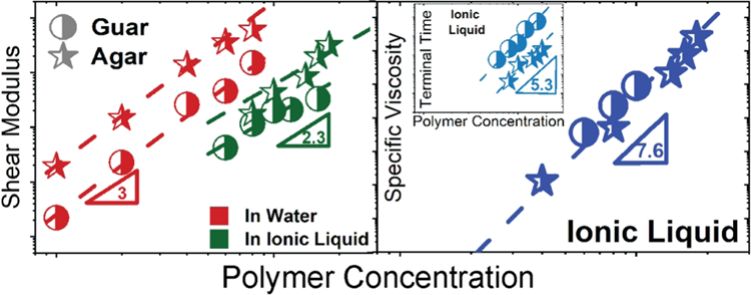

This study investigates the rheological behavior of two plant-based polysaccharides, with different degrees of hydrophilicity, agar (highly hydrophilic) and guar gum (hydrophilic), in water and 1-ethyl-3-methylimidazolium acetate (EMImAc). The rheological response of these polymers is highly dependent on the solvent’s ability to disrupt intermolecular associations. In water, agar forms hydrogels, while guar gum behaves as a viscoelastic liquid with slow modes. The plateau modulus (*G*_N_^0^) scales with polymer concentration (*c*) as *G*_N_^0^ ∼ *c*^3^, consistent with other natural polymers. In EMImAc, both polysaccharides form viscoelastic liquids, exhibiting *G*_N_^0^ ∼ *c*^2.3^, as expected for semiflexible polymer solutions. However, the terminal relaxation time, τ_D_, and the specific viscosity, η_sp_, scale as τ_D_ ∼ *c*^5.3^ and η_sp_ ∼ *c*^7.6^, indicative of intermolecular chain–chain associations. Despite the solvent or polysaccharide, the fractional viscosity overshoot and the shear strain at the maximum stress show a terminal Weissenberg number dependence similar to other synthetic polymers.

## Introduction

Polysaccharides are biopolymers derived from nature. Due to their widespread abundance, they have been seen as a sustainable option to make materials. Biopolymers can be treated using various techniques and they can be formulated into solutions or gels under various conditions. These materials can be employed in numerous domains: food, biomedical, pharmaceutical, cosmetic, and chemical industries.^1−3^ The kind of polysaccharide employed, with its unique molecular structure, and the dissolving media determine the interactions among the polymer chains and consequently, the macroscopic rheological response. Hence, these features can be leveraged to design high-performance products, optimize their processing technique, and devise pertinent process equipment.^1,2,4^

Two such polysaccharides, agar and guar gum, are classified as generally recognized as safe (GRAS) materials due to their suitability for human consumption.^5,6^ They are greatly researched owing to their gelling and thickening capabilities.

Agar is a blend of polysaccharides obtained from marine organisms like red algae and various seaweeds.^7^ They are found in the cell walls of these creatures supporting them to endure the waves and aiding in their biological functions.^8^ The biopolymer can be easily extracted using a simple technique, making it cost-effective and environmentally friendly due to its biodegradable nature.^9^ Owing to its gelling and thickening properties, it is already extensively being employed in the food sector for confectionery, and dairy products. Additionally, it is also widely employed in the microbial studies and bioprocessing sector as culture media.^8^ Furthermore, it has been widely researched for various functions namely tissue engineering,^10^ three-dimensional (3D) printing,^11^ and packaging material.^12^

As shown in Figure S1A, the chemical structure of agar consists of two fractions–agarose and agaropectin. Agarose, the principal component of agar, is a neutral polymer comprising monomeric units d-galactose and 3,6-anhydro-l-galactose connected by β-1,3- and α-1,4-glycosidic bonds, respectively. Agarose is primarily responsible for the gelling properties of agar. On the other hand, agaropectin shares a similar backbone structure, but approximately 8% of the 2- or 6- positions of the 3,6-anhydro-l-galactose contain substitutes, namely methoxyl, sulfate, or pyruvate residues.^13^ Agar is well-known to be soluble in water at higher temperatures and form gels at very low concentrations.^5^

Over the years, researchers have been interested in the rheology of agar in water, especially its thermo-rheological properties.^7,14−19^ Labropoulos et al.^15,16^ developed a detailed theoretical model using shear rheology to understand the gelation mechanism of agar. At higher temperatures, agar chains adopt a random coil shape and below a characteristic gelation temperature (38–45 °C) associate with helical structures that cluster to form other supramolecular structures to give rise to a three-dimensional cross-linked network.^15−17^ Rheological studies aid in assessing the internal microstructure of the agar gel material. Reports state that the mechanical spectra typically exhibit solid-like behavior (with *G*′ > *G*″) with almost frequency independence and the material exhibits higher yield strain values as evidenced from large amplitude oscillatory shear (LAOS) experiments, both implying the completely cross-linked nature of the gel.^7,20−22^ Consequently, flow curves exhibit a strong shear-thinning nature with power law behavior.^20^

Guar gum is a polysaccharide belonging to the family of galactomannans.^23^ Among this family, the polymer backbone comprises mannose monomeric units connected with each other by β-1,4-glycosidic bonds. At different intervals, the backbone possesses grafts of galactose units connected by α-1,6-glycosidic linkages. The primary difference among different polysaccharides found in the family arises due to the varying mannose to galactose (M/G) ratio.^2,23−25^ Their dissolution in water depends on the M/G ratio with a larger ratio demanding higher temperatures. Guar gum obtained from the endosperm of the guar bean (*Cyamopsis tetragonoloba*) has a M/G ratio varying between 1.5–2.0 implying that 1 out of every 2 to 3 mannose monomers is unsubstituted.^26−29^ The structure of guar gum is depicted in Figure S1B. Other biopolymers falling under galactomannans include tara, locust bean, fenugreek, and cassia gums.^23,30^

The guar gum is an economically viable natural polymer owing to its ease of extraction from the guar seeds and widespread accessibility.^31^ Hence, they have been commercially employed as thickeners, and stabilizers in various domains: food, petroleum, agricultural, cosmetic, medical, mining, paper, and textile.^1,2,25^ Similar to agar, guar gum has also been researched for diverse applications in tissue engineering,^32^ drug carrier agents, water purification, and pharmacotherapy.^33^

The rheological properties of aqueous guar gum solutions have been previously studied. An earlier study by Ross-Murphy^34^ reported that the mechanical spectra of aqueous guar gum behave like entangled polymer solutions. Similar dynamic oscillatory responses were also reported by other authors.^1,2,35,36^ Many subsequent articles reported that the flow curve of guar gum/water systems displays non-Newtonian, shear-thinning behavior with viscosity primarily influenced by molecular weight. Also, it is known that the interactions between the chains are dependent on the M/G ratio.^1,2,25,35−38^ Additionally, at lower concentrations, the scaling exponents of specific viscosity, η_sp_ scales with power of 1.2–1.4 with the product of concentration and intrinsic viscosity, *c*[η] (coil overlap), analogous to randomly coiled polysaccharides and synthetic polymers.^37,39^ Interestingly, beyond the entanglement concentration, the scaling law reported varies between 3.4 and 4.0 to 5.1.^37,39^ Consequently, it was realized that the viscosity has a strong dependence on polymer content surpassing values predicted by traditional volume occupancy theories.^29,39^

Interestingly, in addition to studies reporting a higher scaling law for the specific viscosity, Richardson and Ross-Murphy^40^ reported the presence of another transition at lower shear rates in the flow curve. The reported scaling implies that the interactions between guar gum chains are not solely controlled by topological restrictions but also by other polymeric interactions. Researchers addressed this phenomenon and linked it to the presence of additional relaxation modes called as “hyperentanglements” that extend beyond the topological disentanglements.^41−44^ Additionally, Wientjes et al.^45^ performed oscillatory shear experiments on this system and observed the presence of two elastic plateaus–one at a higher frequency and the other at a lower frequency, the latter associated with hyperentanglements.

The complex structure and the hydrophilicity of the polysaccharides control their interactions in water which in turn determines their macromolecular assemblies. Within agarose, the OH-4 of the d-galactose forms a bond with the neighboring hemiacetal oxygen atom of 3,6-anhydro-l-galactose. This intramolecular bonding is enabled owing to the flexibility of the glycosidic bondage between these two units. Additionally, 3,6-anhydro-l-galactose contributes to the rigidity of the chain due to its cage-like structure. Intermolecular hydrogen bonding might occur between the ring oxygen atom at positions 3 and 6 and the hydroxyl group at position 2, which is axially oriented and less flexible, of the 3,6-anhydro-l-galactose units on different molecules. Consequently, in addition to the intra and intermolecular hydrogen bonding, owing to the unique structure of the 3,6-anhydro-l-galactose units, water molecules can bind in a three-dimensional fashion forming a gel.^46,47^ However, guar gum on the other hand is not hydrophilic as agar. Guar gum has a smaller number of interactive hydroxyl groups as compared to agar.^48−50^ Additionally, agar possesses a linear architecture that leads to a more organized arrangement of hydroxyl groups leading to effective network formation with water.^47^ However, guar gum with its branched moieties has a more complex structure with limited accessibility of the hydroxyl groups to water and possess flexible glycosidic linkages thereby favoring more intramolecular interactions than intermolecular.^47−50^ As a result, agar, with its molecular structure that facilitates hydrogen bonding, forms a gel, whereas guar gum, due to its lower hydrophilicity, does not.

Quite recently, ionic liquids (ILs) have been gaining attention as effective solvents owing to their capability to dissolve polysaccharides, especially cellulose.^51−56^ They are liquid salts at room temperature, consisting of two fundamental components: a cation and an anion. The tunability of the kind of ion reveals a plethora of ILs with desired selective dissolution and other useful functionalities.^57^ ILs predominantly aid in the dissolution of polysaccharides through hydrogen bonding (H-bond) competition by overcoming the material’s innate H-bonds.^51,58^

Over time, researchers have conducted various studies to fully understand the cellulose dissolution in IL.^59−64^ An extensive collection of experimental, rheological, and computational results is reported elsewhere.^52−54,56,65−71^ However, much of the fundamental research has been restricted to cellulose in various ionic liquids opening avenues to explore other polysaccharides. Variability of biopolymers in different IL media would result in different interactions in the system, and consequently diverse rheological behavior.^72−74^

Horinaka et al.^75,76^ studied the rheological properties of agarose and guar gum in 1-butyl-3-methylimidazolium chloride (BMImCl). The study showed that the polymers obeyed the time–temperature superposition principle, and characteristic parameters, namely the molecular weight between entanglements (*M*_e_) in the melt state and the number of monomeric units between consecutive entanglements, were determined. They were the first to report such characteristic values of these polysaccharides using ILs, which are otherwise difficult to obtain when dissolved in water.^75,76^ Sharma et al.^77^ used 1-ethyl-3-methylimidazolium chloride and 1-octyl-3-methylimidazolium chloride as cosolvents in agar hydrogels to tune their rheological properties. It was shown that with the addition of the IL, the gelation and melting temperatures of the hydrogels were shifted by 10 °C. Additionally, the IL molecules induce flexibility in the material, and the gel strength can be tailored by varying its composition in the solvent.^77^

Interestingly, guar gum in BMImCl, in combination with other components, has been extensively studied for its mechanical properties.^78−85^ Mine et al.^78^ obtained gels with guar gum/BMImCl which was converted to thermosensitive shape-changing films. Sharma et al.^79,80^ developed self-healing gels with high strength by incorporating multiwalled carbon nanotubes in guar gum/BMImCl. Zhang et al.^81^ combined guar gum with BMImCl and an imidazolium-based poly(ionic liquid) to develop thermally stable, elastic, and conductive gels.

Since the rheological response of the two aforementioned plant-based polymers, among others, strongly depends on the solvent’s effectiveness in disrupting intermolecular polymer chain associations, it is valuable to provide a direct rheological comparison, using biopolymers with different degrees of hydrophilicity, same mass fractions, and two distinctly different solvents: water and 1-ethyl-3-methylimidazolium acetate (EMImAc).

The dynamics of these polysaccharides were assessed by using small amplitude oscillatory shear (SAOS) rheology, whereas start-up of shear experiments were employed to delineate the complex nonlinear behavior of the polysaccharides, pivotal for processability. Scaling laws for modulus, terminal relaxation time, specific viscosity, and nonlinear rheological functions were obtained and contrasted to those known for synthetic semiflexible chains. In addition, a recent molecular model, namely sticky-blob,^86^ was invoked to explain the associating polymer properties exhibited by agar and guar gum dissolved in EMImAc. We show that by leveraging the unique properties of the ionic liquid, it is possible to alter the polysaccharide solubility, and the chain–chain interactions, thereby controlling material properties such as mechanical strength and viscosity. This level of control opens new pathways for designing advanced polymeric materials with tailored functionalities for specific applications.

## Materials and Methods

### Preparation of Polysaccharide Solutions

Agar powder CMN (courtesy of Kamperman Lab, University of Groningen) was procured from Boom Lab (CAS number 9002–18–0, batch number PROD2102326). Guar was obtained from Sigma-Aldrich (CAS number 9000–30–0, product number G4129). All polymers were used as received. The ionic liquid (IL) solvent 1-ethyl-3-methylimidazolium acetate (EMImAc) was purchased from abcr GmbH (Karlsruhe, Germany) with a purity of ≥95% and CAS number 143314–17–4 and used without any further purification. The polysaccharide/IL solutions were prepared by adding the required amount of polymers in EMImAc and maintaining them at 80 °C for 30 min with gentle stirring. Concentrations ranging between 1 and 14 wt % were prepared. Similarly, the aqueous solution of these polysaccharides was prepared in a similar fashion by adding the appropriate amount of polymers to Milli-Q water with gentle stirring at 80 °C for 30 min. Concentrations ranging between 1 and 8 wt % were prepared.

### Rheometry

The rheological experiments were carried out with a Discovery Hybrid Rheometer (HR-2) from TA Instruments (New Castle) with a parallel plate configuration. In accordance with the viscosity of the samples, 8-, or 25 mm diameter plates were utilized. Polysaccharide/EMImAc solutions were loaded into the rheometer at 80 °C and annealed for 30 min to erase the thermal history and eliminate any moisture present in the sample. Consequently, the temperature was reduced and after thermal equilibration, the measurements were performed at 35 °C. The polysaccharide/H_2_O gels were loaded and measured directly at 35 °C. To prevent water evaporation, the sample was surrounded by a nonimmiscible oil. The temperature was controlled via an electric heater.

### Linear Rheology

Small amplitude oscillatory shear (SAOS) measurements were employed to study the linear viscoelastic (LVE) regime of the systems in both solvents. The sample was loaded into the rheometer, surrounded by oil (for aqueous solutions) and before any measurement, it was subjected to a rejuvenation and aging protocol. The rejuvenation is a dynamic time sweep (DtS) at 1 rad/s and a selected 200% strain (ensured to choose a strain falling in the nonlinear regime by performing a dynamic strain sweep (DSS) at 1 rad/s), typically for 60–100 s (until steady state was observed) to erase the mechanical history of the sample. Subsequently, the aging is a DtS conducted at 1 rad/s and strain falling in the LVE regime for 200 s to allow the system to attain a steady structure (see Supporting Information). Additionally, DSS experiments were performed at 100 rad/s to ensure that the LVE regime applies to the whole frequency spectrum probed. Subsequently, dynamic frequency sweeps (DFS) were performed over a frequency range of 100 to 0.01 rad/s. For the gel-like samples exhibiting no frequency dependence, the DFS was carried out from 100 to 0.1 rad/s.

### Nonlinear Rheology

The nonlinear rheology of the polysaccharides in different solvents was explored through start-up of shear experiments. The material was subjected to shear rates varying from 0.01 to 100 s^–1^ at 35 °C. The shearing time was chosen to ensure that steady-state values were attained at each shear rate. However, for the polysaccharide/EMImAc samples exhibiting a crossover in the LVE, 5 min (>10 × τ_D_, where τ_D_ is the terminal crossover time) waiting time was observed prior to applying each shear rate. This was done to ensure that the material fully relaxed before proceeding to next measurement. The determined flow curves were corrected by applying the Weissenberg–Rabinowitsch correction factor^87^ to account for the radial shear rate dependence in the parallel plate geometry
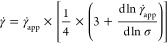
1where γ̇ is the corrected true shear rate, γ̇_app_ is the apparent shear rate, and σ is the shear stress.

## Results

### Linear Viscoelasticity

**Figure 1:**
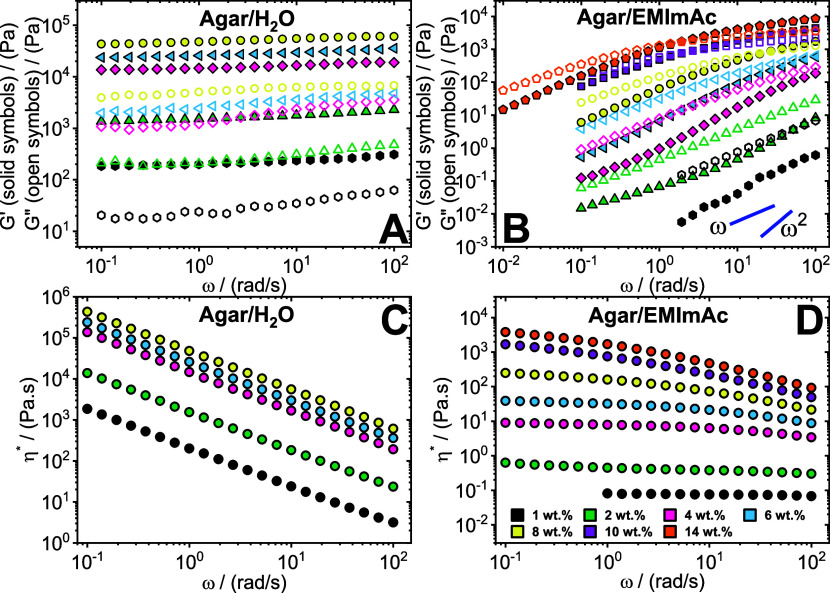
Linear viscoelasticity of agar. Storage *G*′ (solid symbols) and loss *G*″ (open symbols) modulus as a function of oscillation frequency ω and the complex viscosity η* as a function of ω for agar dissolved in water (A, C) and in EMImAc (B, D) across various mass fractions (see legend). Experiments were performed at 35 °C. The blue lines in panel (B) represent the terminal slopes of a fully relaxed viscoelastic liquid.

To assess the effect of solvent on the rheological response in the studied polysaccharides, small amplitude oscillatory shear (SAOS) experiments were employed. Figure 1 depicts the dynamic frequency responses for agar dissolved in water and EMImAc. It can be observed from Figure 1A (black data points) that at lower concentrations of 1 wt %, agar dissolved in water shows a solid-like response with (*G*′ > *G*″) with almost no angular frequency dependence. Quite recently, El-hoshoudy and co-workers^88,89^ explored the rheology of gels prepared with composites of chitosan^88^ and xanthan gum.^89^ They observed that the material exhibited moduli values of *G*′ > *G*″, thus signifying the gel nature of their material with higher amount of cross-linking and consequently, larger ability of the system to store mechanical energy. In congruent to the team’s results, we observe that our rheological response (in Figure 1A) resembles that of a gel.^34,90^ Upon increasing agar content up to 8 wt % in water, the response remains unaltered but moduli increase in value; stronger gels are formed.

The gelation of agar in water has been a widely researched topic.^7,46,90−98^ It is commonly debated whether agar gelation involves either spinodal decomposition or conformational change of the polymer chains.^96^ This results in a cross-linked network with the agarose chains adopting helical conformation.^92^ It was reported that helices play a role in creating the junction zones required for the formation of the gel network.^93^ Upon cooling, these higher-order structures coalesce to generate bundles, leading to the establishment of robust gel structures.^7^ Consequently, these structures result in high and frequency-independent moduli, as observed in Figure 1A.

On the contrary, agar in EMImAc (Figure 1B) exhibits completely different dynamics as opposed to water. First, it can be observed that at a low agar concentration of 1 wt % (black data points) in EMImAc, the moduli values are significantly lower compared to those in water. Second, the loss modulus *G*″ is much higher than storage modulus *G*′ implying the liquid-like nature of the solution. Consequently, we can also notice the strong dependence of the moduli values on the angular frequency. Hence, agar in EMImAc results in a much more dissolved state of the polysaccharide than in water, giving rise to a viscoelastic liquid.

Furthermore, upon increasing the agar concentration in EMImAc, up to 6 wt %, the system remains dominatingly dissipative (*G*″ > *G*′) with a strong dependence of moduli on the frequency. Interestingly, in contrast to water, concentrations much higher than 8 wt % were attainable in EMImAc. Additionally, it can be observed from Figure 1B at 8 wt % (yellow data points), that the system exhibits a distinct terminal crossover in the frequency window studied. It is conjectured that this type of rheological response resembles that of an entangled polymer solution, whose terminal time refers to the time scale at which reptation events occur.^99^ As expected, moving to higher concentrations of 10 and 14 wt % (purple and orange data points), the crossover shifts to lower frequencies illustrating the slowing down of the polymer dynamics at higher polymer content, as a result of a larger number of entanglements formed in the system.

The differences in the solvent-controlled rheological response of agar in water and EMImAc can be also observed by comparing the complex viscosity reported in Figure 1C, and 1D, respectively. As previously mentioned, agar in water always forms gels in the concentration regime explored, therefore the complex viscosity only exhibits a shear thinning behavior in the whole frequency range probed (see Figure 1C). Note also that, the magnitude of the low-frequency viscosity values range from 10^3^ to ∼10^6^ Pa·s, from 1 to 8 wt %. On the contrary, when EMImAc is used as a solvent, weak shear-thinning fluids with clear Newtonian behavior at low frequencies, are obtained. Remarkably, the magnitude of the viscosity is much lower than the samples prepared in water at the same mass fraction, even by several orders of magnitude (see for instance 1 wt % black data in Figure 1C, and 1D). This is a robust indication that EMImAc is very effective in disrupting the complex structures formed by agar in water.^92^ As a result, agar/EMImAc solutions, exhibit characteristics similar to semiflexible polymer solutions, featuring a semidilute unentangled and a concentrated entangled regime, with the presence of an entanglement plateau and terminal/disentanglement time. However, as it will be shown later in the text, EMImAc does not prevent all the intermolecular associations.

**Figure 2:**
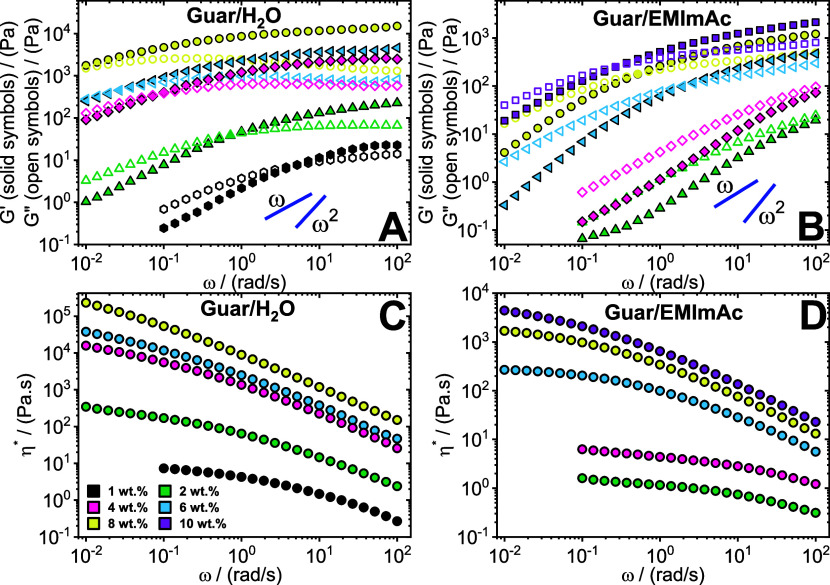
Linear viscoelasticity of guar gum. Storage *G*′ (solid symbols) and loss *G*″ (open symbols) modulus as a function of oscillation frequency ω and the complex viscosity η* as a function of ω for guar gum dissolved in water (A, C) and in EMImAc (B, D) across various mass fractions (see legend). Experiments were performed at 35 °C. The blue lines in panels (A, B) represent the terminal slopes of a fully relaxed viscoelastic liquid.

Figure 2 portrays the SAOS behavior of guar gum dissolved in water and EMImAc. Unlike agar, guar gum in water displays the characteristic of viscoelastic liquids (Figure 2A); unentangled or entangled systems, depending on the concentration, with the presence of an entangled plateau and a terminal moduli crossover. The reason for this difference lies in the lower hydrophilicity of guar gum compared to agar. Their branched structure limits the accessibility of water molecules to their hydroxyl group and the flexibility of the glycosidic linkages between their monomers favors intramolecular bonding preventing the formation of any three-dimensional network.^47^ Interestingly, the slopes of the moduli in the terminal regime do not exhibit the expected terminal slopes of 2 and 1 for *G*′ and *G*″, respectively, for fully relaxed fluids. Consequently, on performing SAOS on 4 wt % guar gum in water (Figure S2) over a very long time (or very low angular frequencies) we noticed a clear crossover to a solid-like response. This gives a clear indication that the system possesses long-time dynamics with complex behavior. The presence of two elastic plateaus has been reported in other works. For instance, it was reported that intermolecular associations between mannose monomeric units (termed “hyperentanglements”) were posited to account for the relaxation processes that extend beyond topological disentanglements.^41,43−45^ Wientjes et al.^45^ were the first to report the presence of a second crossover at lower frequencies for guar gum in water through SAOS measurements. The team posited that conventional reptation theory cannot explain the observed behavior and it demands two or more relaxation mechanism theories. Their work claimed that the system either possesses two kinds of associating stickers responsible for their dual crossover or star-like structural relaxation due to significant physical bonds.^45^ Therefore, guar dissolved in water depicts complex polymer dynamics with diverse relaxation mechanisms resulting in their double elastic plateau.

When guar gum is dissolved in EMImAc, the frequency response differs from that obtained in water solutions, as shown in Figure 2B. It can be observed that 2 wt % guar in EMImAc (green data points) displays fully viscous behavior (with *G*″ > *G*′) and interestingly, moduli values lower than 1 wt % guar in water. As we increase guar gum content in EMImAc, e.g. at 6 wt % (blue data points), a moduli crossover indicating the inverse of a disentanglement time manifests. As expected, upon increasing guar gum concentration, the crossover shifts to lower frequencies and raises the moduli values. Additionally, upon performing SAOS on guar gum in EMImAc solution over a very long time (Figure S3), the material demonstrated the terminal slopes of 1 and 2 and did not exhibit any other discernible crossover at lower frequencies. Thus, the dissolution of guar gum in EMImAc aids in preventing hyperentanglements and consequently, averting complex dynamic behavior that is otherwise observed in water.

For the same mass fraction of guar gum, the moduli values are much lower when dissolved in EMImAc compared to water. The difference can be evidently observed by comparing the complex viscosity values between Figure 2C, and 2D. On comparing the data points (green, pink, blue, and yellow) between the two panels, almost 2 orders of magnitude difference can be observed for guar gum dissolved in water over EMImAc.

**Figure 3:**
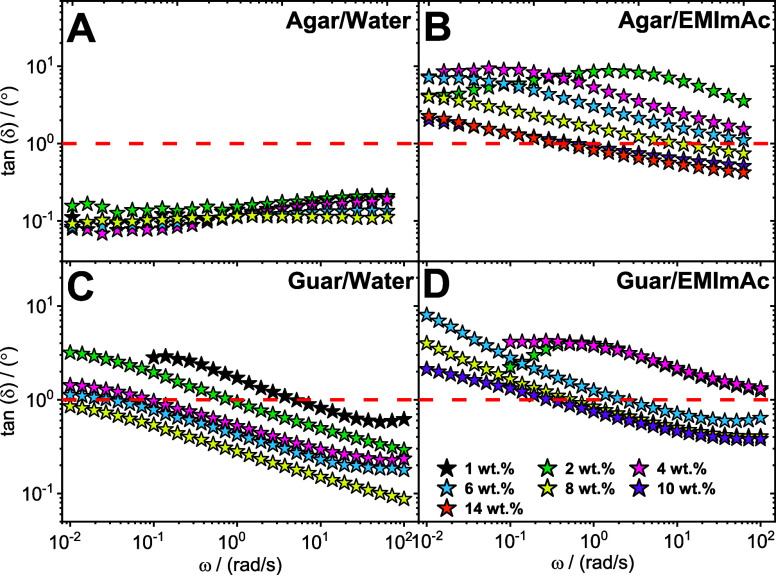
Dynamic phase behavior of polysaccharide in different solvents. Loss factor tan(δ), derived from SAOS, expressed as a function of oscillation frequency ω for polysaccharides dissolved in water and EMImAc across different polymer contents (see legend). (A, B): Agar and (C, D): guar. Experiments were performed at 35 °C. The red colored dashed line in all the panels marks the tan(δ) value of 1 and for solutions with tan(δ) < 1 predominantly exhibits an elastic response while those with tan(δ) > 1 exhibits a majorly viscous behavior.

The dynamic phase behavior of agar and guar gum in water and EMImAc can therefore be analyzed by plotting the loss factor (tan(δ) = *G*″/*G*′) as a function of the angular frequency (Figure 3). The dashed red line across all the panels corresponds to tan(δ) = 1; solutions with a loss factor less than 1 exhibit an elastic-dominated response, whereas systems with tan(δ) > 1 are highly dissipative (viscoelastic liquids).

Panel 3A depicts the loss factor for agar in water. The tan(δ) values are well below the red line across all concentrations and frequency ranges. They do not exhibit any dependence on frequency whatsoever. Similar tan(δ) values and their frequency independent behavior were reported for other biopolymer gels.^88,89^ It is known that agar is composed of agarose which can strongly associate through H-bonding.^46,95^ This results in a cross-linked network with the agarose chains adopting helical conformation.^92^ It was reported that helices play a role in creating the junction zones required for the formation of the gel network.^93^ With cooling, these higher order structures coalesce to generate bundles, leading to the establishment of robust gel structures.^7^ Consequently, these bundles can withstand great stresses making them elastic in nature and giving rise to their low tan(δ) values seen in Figure 3A.

On the contrary, when agar is dissolved in EMImAc, the loss factor across all concentrations tends toward values greater than one (Figure 3B). It is well-established the dissolution capacity of IL toward polysaccharides.^66,72^ Kumar and team^100^ studied the conformational change of agarose when dissolved in different ILs through circular dichroism (CD) spectroscopy. Agar dissolved in 1-butyl-3-methylimidazolium chloride, *N*-butyl-3-methylpyridinium chloride, and 3-methyl-1-octylimidazolium chloride exhibited significant shift and loss of intensity in the spectra as compared to native agar. The results were indicative that dissolution of agar in ILs could lead to possible change in conformation and disruption of their higher ordered structures.^100^ Agar when dissolved in IL loses their helical cross-linked network (seen when in water) and results in lower-ordered structures. Therefore, the system remains predominantly viscous ensuring the high tan(δ) values seen in Figure 3B.

When guar gum is dissolved in water, viscoelastic liquids are formed, and the tan(δ) shows solid-like behavior at high frequencies due to the presence of entanglements (Figure 3C) and liquid-like response at low frequencies, when reptation events occur. This rheological response is in agreement with many works reported in the literature.^1,34,35,101−103^ However, as seen in the earlier discussion, at higher concentrations, there exists the presence of “hyperentanglements” where the mannose monomeric units aggregate through intermolecular interactions making the picture more complex.^41,44,45,104,105^ In a recent work by Liang et al.,^29^ it was postulated that the rheological behavior of guar gum/water can highly vary based on the M/G ratio, arrangement of the mannose units that consequently affects their aggregation. Nevertheless, based on the results in Figure 3C, we observe the guar gum chains participate in strong intermolecular interactions and as evidenced by Figure S2, there is a clear presence of “hyperentanglements”.

However, when we dissolve guar gum in EMImAc, as shown in Figure 3D, the loss factor values are greater than one in the same frequency spectrum. Charlot and co-workers^106^ studied the solubility of guar gum in various ILs, including EMImAc, and characterized their interactions. Through investigations with Fourier Transform infrared spectroscopy (FTIR) techniques, it was shown that when guar gum was dissolved in EMImAc, there was a significant increase in the H-bonding strength, i.e., the formation of H-bonding interactions between guar gum chains and EMImAc. Additionally, it was posited the IL ions serve as a “chaotropic agent” in the solution. This implies that the ions physically obstruct the H-bonding sites on the polymer backbone, diminishing interactions between the guar gum chains, and leading to improved dissolution of guar gum. Moreover, the activation energy of guar gum in water and EMImAc were reported to be 10 and 43.9 kJ/mol, respectively. The activation energy in EMImAc is almost four times greater than that in water corresponding to IL/IL electrostatics, van der Waal forces, H-bonding among the ions, and between the guar chain and IL molecules.^106^ Consequently, the greatly dissolved state of guar gum makes the system highly dissipative giving rise to their tan(δ) values shown in Figure 3D. Additionally, EMImAc prevents the formation of aggregates which is further supported by the absence of “hyperentanglements” as shown in Figure S3.

**Figure 4:**
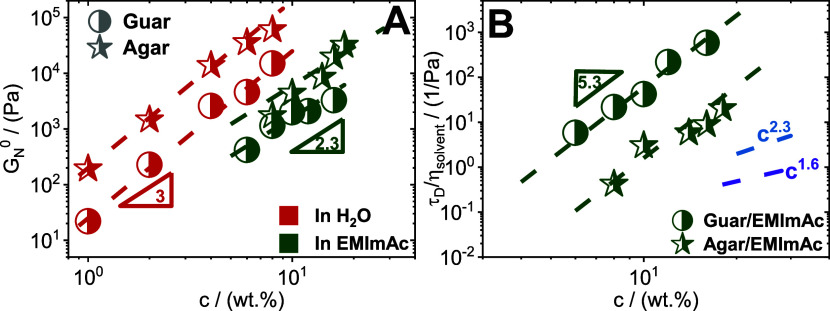
Concentration dependence. (A) Plateau modus *G*_N_^0^, determined at the minimum of tan(δ) from the SAOS measurements, plotted as a function of polymer concentration *c* for agar and guar gum dissolved in water and EMImAc at 35 °C. The dark red and dark green dashed lines correspond to a slope of 3 and 2.3 between storage moduli and polysaccharides content in water and EMImAc, respectively. (B) Correlation between the longest relaxation time, τ_D_, determined as inverse of crossover frequency from the SAOS measurements, divided by solvent viscosity as a function of polymer content for polysaccharides dissolved in water and EMImAc at 35 °C. Slope of 5.3 discussed in the main text is illustrated here as dark green dashed lines for polysaccharides in EMImAc. Slopes of 2.3 and 1.6 pertaining to the dependence of the relaxation time on concentration for semiflexible nonassociating polymer chains in θ and good solvency conditions are depicted as blue and purple dashed lines, respectively.

The plateau modulus (derived from the linear viscoelastic spectrum as the storage modulus value at the point where tan(δ) = *G*″/*G*′ reaches its minimum) plotted as a function of polysaccharide concentration is shown in Figure 4A. Both polysaccharides in water exhibit a plateau modulus that grows with a power law of 3. It is established that for entangled polymer solutions, there exists the relationship of *G*_N_^0^ ∼ *c*^*x*^, where *G*_N_^0^ is shear modulus, *c* is the concentration of polymer, and *x* is 2.25 for good solvency condition and 2.33 for θ solvent condition.^107^ It is noteworthy to observe the deviation of plateau modulus to higher scaling values in water. In the work of Clark and Ross-Murphy^108^ on concentration dependence of gelatin and agar hydrogel modulus, a similar scaling law of *G*_N_^0^ ∼ *c*^3^ was observed. The most suitable interpretation could stem from the arrangements of molecules that make up the three-dimensional networks.^109^ The elasticity (and consequently, shear modulus) of a polymeric system stems from the entropic stretching of the chains.^99^ Even so, in the case of a polymer with rigid monomers, the enthalpic bending of strands becomes a contributing factor to elasticity. Consequently, it is vital to consider the backbone of the polymeric chain, and typically, polysaccharides are more rigid than synthetic polymers.^109^

Kim et al.^109^ studied the contribution of chain stiffness to the elastic modulus of biopolymer systems. They explained that when the polymer segments between two points of contact exhibit flexibility, the movement of the interconnected segments becomes fully dissociated. In contrast, rigid segments exhibit a cooperative motion dragging multiple segments. This implies that deforming rigid connections requires a significantly higher force compared to inducing deformation in flexible strands. Consequently, agar and guar gum, with their innate hydrogen bonds in water possess much stiffer backbones making the dependence of shear modulus on their concentration greater than the conventional scaling laws.^109^

Contrarily, as seen in Figure 4A, both polysaccharides, when dissolved in EMImAc, exhibited a dependence of 2.3 and lower modulus values than when dissolved in water, implying that the solvent can disrupt the extensive H-bonds present in these systems, preventing supramolecular structures.

However, a noteworthy observation from Figure 4A is that the scaling law is reduced, as opposed to water. This suggests that EMImAc disrupts the interactions between chains and possibly decreases their persistence length and, consequently, "altering" the flexibility of the backbone. Hirosawa et al.^110^ in their work on the structural configuration of cellulose in phosphonate-based ionic liquids, discussed the changes in the flexibility of the polymer with varying solvents. They compared results from other studies and reported that the flexibility of the biopolymer conformation depends on the solvating capability of the ionic liquid employed.^110−112^

To further understand the effectiveness of the ionic liquid in dissolving the polysaccharide chains, the polymer concentration dependence of the terminal relaxation time of the polymers was assessed (Figure 4B). Herein, the chain topological disentanglement time, τ_D_ (determined as the inverse of the crossover frequency from the SAOS measurements), divided by the solvent viscosity, is plotted against the polymer content for both polymers in EMImAc. It was found that τ_D_ ∼ *c*^5.3^, reflecting intermolecular chain associations in θ solvency conditions.^86,99^ Importantly, semiflexible nonassociating polymer chains would exhibit τ_D_ ∼ *c*^2.3^ in θ solvency and τ_D_ ∼ *c*^1.6^ good solvency conditions.^54,99^ Thus, it is evident that EMImAc suffices in overcoming a significant number of H-bonds, thus preventing the formation of supramolecular structures, i.e., helices or bundles in the case of agar, and aggregates in the case of guar gum. However, EMImAc does not prevent intermolecular chain–chain associations and thus exhibits dynamics typical of semiflexible associating polymers. This aspect will be further discussed in the next section.

### Nonlinear Shear Rheology

The nonlinear viscoelastic shear response of the polysaccharides dissolved in water and EMImAc was investigated through start-up of shear experiments. Particular emphasis is given to the so-called Cox–Merz rule; an empirical equivalence between the complex viscosity determined by dynamic oscillatory measurements and the steady-state shear rate-dependent apparent viscosity of polymers. With the aid of this empirical relationship, steady shear properties of a material can be determined from the oscillatory dynamic measurements.^113^ Note that, capturing the steady-state viscosity of many polymeric systems is challenging, especially at higher shear rates, due to flow instabilities, such as edge fracture and secondary flows.^86,114^ Thus, the Cox–Merz rule has proved a useful and reliable tool in determining the material properties under flow which are consequently vital for their optimal processing in industries.

**Figure 5:**
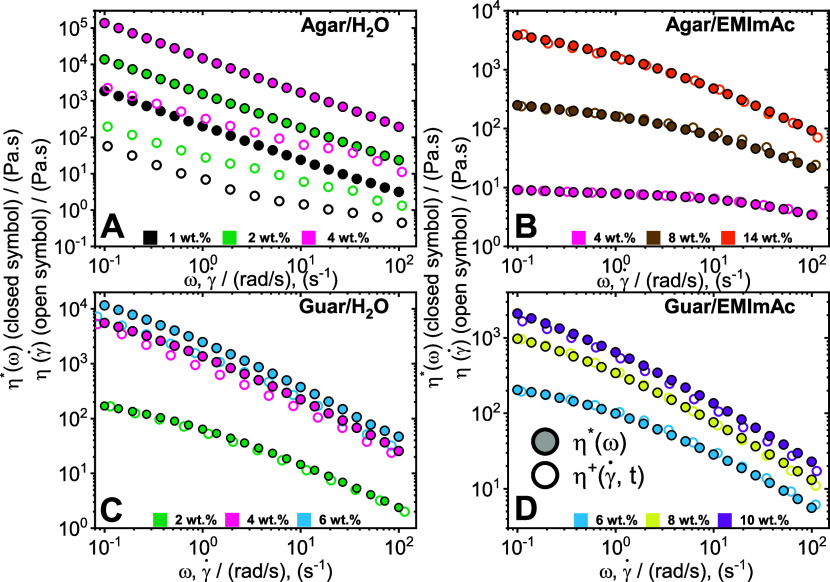
Cox–Merz applicability for polysaccharides in different solvent. Steady shear viscosity (open symbols) from shear start-up measurements and complex viscosity (closed symbols) from SAOS plotted with respect to each other to determine the pertinence of Cox–Merz rule at 35 °C for agar dissolved in water and in EMImAc (panels A, B, respectively), and guar gum dissolved in water and in EMImAc (panels C, D, respectively) across different concentrations (see legends).

In Figure 5, the Cox–Merz validity has been tested for agar and guar gum in different solvents. Across all panels, closed symbols depict complex viscosity whereas open symbols represent the steady-state shear viscosity determined from start-up of shear experiments. The stress growth coefficient of agar dissolved in water and EMImAc is reported in Figures S4 and S5, while for guar gum, it is noted in Figures S6 and S7, respectively.

In Figure 5A, the data points follow a power law behavior indicative of a greatly shear-thinning material. Across the three concentrations explored, we see substantial deviation of the viscosity upon shear. This departure from the Cox–Merz rule is indicative of a highly elastic gel-like network. Similar deviations have been reported for other polysaccharides namely, gellan, schizophyllan, and xanthan gum that show gelation in water.^115−118^ As we have already seen in the previous section, agar possesses higher-order structures owing to its helices that form bundles. The network is not disrupted during the small amplitude oscillatory measurements, however upon shear, these bundles can be broken down leading to significantly lower viscosity.^119^ Additionally, it can be observed that as we go to higher concentrations (pink data points), the deviation is much higher (almost two decades) as compared to lower concentrations (black data points). This is a further confirmation that on increasing agar content in water, the higher order structure gets further strengthened and the system becomes more elastic.

In Figure 5B, we observe the Cox–Merz validity of agar in EMImAc. Interestingly, we see a great contrast compared to the behavior from Figure 5A and the values are considerably lower when dissolved in EMImAc. Ranging between concentrations of 4 to 14 wt %, we see almost perfect overlap of the apparent viscosity with complex viscosity. The solvent EMImAc plays a significant role in disrupting the H-bonds and preventing the formation of complex superior structures. Hence, as seen during the SAOS measurements, the system remains highly dissipative without any network. Consequently, we see that the application of shear does not lead to any drop in viscosity and the system behaves like a typical polymeric solution obeying the Cox–Merz relationship.

The Cox–Merz rule applicability in guar gum/water systems can be seen in Figure 5C. It is possible to observe that at low concentration of 2 wt % (green data points), there is a fair overlap, and the system complies with the Cox–Merz rule. Analogous agreement with the Cox–Merz rule has been reported at low concentrations of guar gum, pectins, tara gum, locust bean gum, and various galactomannans.^1,34,120−122^ However, at higher guar contents we see that the steady shear viscosity departs from the complex viscosity. Additionally, the deviation is not severe as the case for agar in water seen in Figure 5A. This result is congruent with reports of guar gum in water at higher concentrations.^1^ In a scattering study by Gittings et al.,^105^ it was reported that guar gum in water forms aggregates of varying sizes depending on polymer concentration. The aggregates were stated to be loosely interlocked with individual chains or other aggregates and are vital for the viscoelastic response of the system.^105^ Consequently, the departure from the Cox–Merz rule in Figure 5C is indicative of the presence of these aggregates that are getting severed upon shear. In addition, the emergence of Cox–Merz deviation at higher polymer concentrations is also in agreement with the results of Gittings et al.^105^ for which it was stated that aggregates were of fractal morphology with sizes varying between 10–100 μm, depending on concentration. Hence, at lower concentrations (green data points), the aggregates are too small to generate the drop in apparent viscosity. At higher concentrations, the aggregate dimensions are big enough to generate an impact upon shear.

Finally, the Cox–Merz applicability was assessed for guar gum dissolved in EMImAc as portrayed in Figure 5D. The foremost observation compared to Figure 5C is the significantly lower viscosity values owing to the greater dissolution power of EMImAc. Both 6 and 8 wt % (blue and yellow data points) show very clear superposition of complex and apparent viscosities implying that the Cox–Merz rule is obeyed. Additionally, even at the high concentration of 10 wt %, there is only a slight deviation of the steady shear viscosity from the apparent viscosity. It is possible at this high guar gum content, the material starts to experience edge fracture causing this deviation. Nonetheless, it is evident the stark difference in the Cox–Merz deviation between Figure 5C, and 5D, and that in water, the deviation starts at a much lower concentration. This suggests that EMImAc severely prevented any formation of aggregates. Hence, it is fair to say that guar gum dissolved in EMImAc reasonably obeys the Cox–Merz rule in the concentration ranges explored.

**Figure 6:**
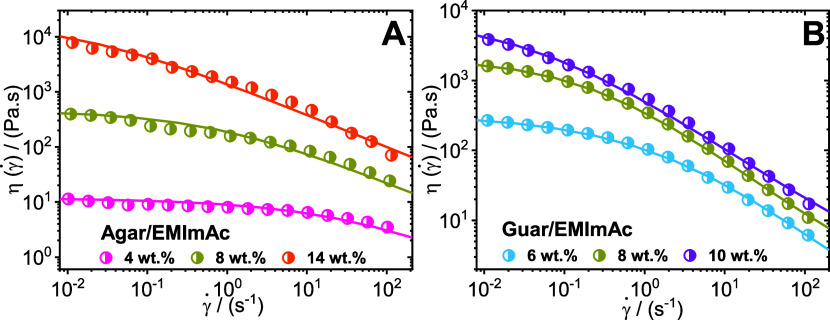
Flow curves of polysaccharides dissolved in EMImAc. Steady shear viscosity obtained from start-up of shear measurements plotted as a function of shear rates for agar (panel A), and guar gum (panel B) dissolved in EMImAc across different concentrations (see legends). The solid lines in the panels represent fitting with Carreau–Yasuda model with the fitting parameters listed in Table 1. Experiments were performed at 35 °C.

To determine the zero-shear viscosity, the solutions exhibiting a Newtonian plateau were fitted to the Carreau–Yasuda model^123^
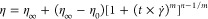
2where η is the steady-shear viscosity, η_0_ is the zero-shear viscosity, η_∞_ is the infinite shear viscosity, *t* is the time constant, γ̇ is the shear rate, *m* is called the transition control factor that describes the transition between Newtonian plateau and power-law region, and *n* being the power index. The flow curves of agar and guar gum dissolved in EMImAc across different concentrations are depicted in Figures 6A, and 6B, respectively. The fitted parameters are enumerated in Table 1.

**Table 1:** Fitting Parameters for the Carreau–Yasuda Model, Equation 2 for Polysaccharides Dissolved in EMImAc (Figure 6)

system	concentration, *c* (wt %)	zero-shear viscosity, η_0_ (kPa·s)	infinite shear viscosity, η_∞_ (Pa·s)	time constant, *t* (s)	transition control factor, *m*	power index, *n*
Agar/EMImAc	4	0.012 ± 0.07	0.003 ± 0.001	0.014 ± 0.11	0.27 ± 0.06	0.6 ± 0.1
Agar/EMImAc	8	0.8 ± 0.2	1.5 ± 0.5	29.3 ± 7	0.3 ± 0.02	0.6 ± 0.02
Agar/EMImAc	14	18.4 ± 2.2	4.7 ± 0.8	74.2 ± 15	0.38 ± 0.02	0.45 ± 0.04
Guar/EMImAc	6	0.3 ± 0.02	0.5 ± 0.2	0.35 ± 0.1	0.43 ± 0.04	0.01 ± 0.03
Guar/EMImAc	8	2.1 ± 0.07	1.8 ± 0.04	4 ± 1.1	0.54 ± 0.04	0.13 ± 0.02
Guar/EMImAc	10	7.9 ± 2.7	2.3 ± 0.1	25 ± 3	0.52 ± 0.02	0.23 ± 0.02

As seen in Table 1, for agar and guar gum, with an increase in polymer concentration, there is a rise in the zero-shear viscosity. The increase in viscosity as the polymer concentration increases implies a larger number of topological interactions among the biopolymer molecules.^1,99,124^ Moreover, a similar incremental trend can be observed for the time constant with an increase in polysaccharide content in EMImAc. The increase implies growth in chain entanglement density. It was posited that the mobility of individual chains is gradually constrained, thereby extending the time required to establish new entanglements, replacing those disrupted by external deformation.^1,99,125^ Hence, the degree of shear-thinning intensifies, and its onset shifts to lower shear rates with increasing polymer concentration as seen in Figure 6.

Interestingly, the value of *m* for agar in EMImAc is much smaller than guar in EMImAc. This is also evident in comparing the panels of Figure 6, where it can be noticed that the extent of shear thinning for guar in EMImAc is significantly greater than that for agar. Additionally, on comparing the polysaccharides at the same mass fraction (yellow data points), guar gum has a higher zero-shear viscosity, but as the shear rate increases, the drop in viscosity is steeper than agar. This trend is a typical feature of branched polymer chains and guar gum possesses short side chain branches (at every galactose unit, see Figure S1) as opposed to agar which is a linear polymer chain.

Consequently, the zero-shear viscosity determined from Figure 6 was utilized to determine the specific viscosity, η_sp_ as follows
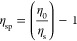
3where η_0_ is the zero-shear viscosity and η_s_ is the solvent viscosity, and its concentration dependence for the polysaccharides in EMImAc was studied.

**Figure 7:**
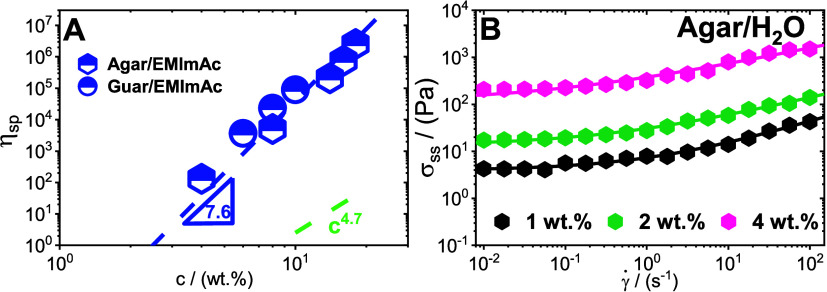
Nonlinearity of polysaccharides in different solvent. (A) Concentration c dependence of specific viscosity η_sp_ of agar and guar gum dissolved in EMImAc. The dashed lines represent a power law with slope of 7.6 indicative of associating polymers. The green dashed lines represent the slope of 4.7 expected for entangled neutral polymers in θ solvency conditions. (B) Steady-state stress σ_ss_ determined from shear start-up measurements as a function of varying shear rates for agar dissolved in water across different concentrations (see legend). The fitting line represents the Herschel-Bulkley fits (eq 5) with the constitutive parameters and yield stress values listed in Table 2.

Figure 7A represents the specific viscosity of agar and guar gum dissolved in EMImAc as a function of polymer concentration. A strong correlation with a power law behavior of ∼7.6 was observed. Note that this value is much higher than that expected for entangled neutral polymers in θ solvency conditions, ∼4.67.^99^ The high dependence of specific viscosity on polymer content in addition to the disentanglement time scaling, τ_D_ ∼ *c*^5.3^, seen in Figure 5B, implies that agar and guar gum behave as strongly associating polymers when dissolved in EMImAc.

In a previous work on the associative properties of poly(vinyl alcohol) solutions,^86^ the sticky-blob model was proposed in order to explain the strong power law dependence of the specific viscosity on concentration. Within the framework of the model, it was reported that for strongly associating entangled polymers in θ solvency conditions^86,126,127^
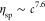
4

Interestingly, the correlation of 7.6 observed in Figure 7A confirms that the sticky-blob model can be applied to agar and guar gum when dissolved in EMImAc.

Based on the model, each entanglement strand contains numerous stickers, and the same holds true for each correlation blob. Consequently, most associations within the blob are intramolecular, given that the blobs essentially reach their overlap concentration, making the interior of the blobs akin to a dilute solution with a single chain. As we have seen, EMImAc behaves as a chaotropic agent for polysaccharides physically interposing beside the polymer chains preventing the formation of higher-order structures. Thus, within each blob, owing to the physical presence of the IL ions, various interactions namely, electrostatics between the IL molecules, the van der Waals interactions, and strong H-bonding between the polysaccharide chains and the IL ions can be expected. Also, as per the model, each sticky blob contributes approximately one intermolecular association which could be the IL ion interacting with two polysaccharide chains simultaneously.

The above explanation can be supported by the work of Charlot and the team^106^ studying interactions between guar gum and EMImAc. It was reported that the activation energy of guar gum is four times greater in EMImAc than when it is dissolved in water, suggesting that this is due to a wide range of associative interactions.^106^ Hence, EMImAc prevents the formation of any supramolecular structures or aggregates, instead, it intercalates between the polymer chains and ensures solubility to a larger extent.

For the gel systems, i.e., agar dissolved in water, the steady-state stress determined from the step rate experiments (Figure S8) was plotted as a function of shear rate in Figure 7B. It is possible to observe that the data points across the three concentrations do not extrapolate to zero stress implying that the system is a yield stress material.^128^ The data points can be reasonably well-fitted to the Herschel–Bulkley equation
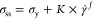
5where σ_ss_ is the steady-state stress, σ_y_ is the yield stress, *K* is the consistency factor, γ̇ is the applied shear rate and *f* is the flow index. All the parameters used for fitting are listed in Table 2.

**Table 2:** Fitting Parameters for the Herschel–Bulkley Model, Equation 5, for Agar Dissolved in Water (Figure 7B)

system	concentration (wt %)	yield stress, σ_y_ (Pa)	consistency factor, *K* (Pa/s^*f*^)	flow index, *f*
Agar/H_2_O	1	4 ± 0.5	3 ± 0.5	0.54 ± 0.03
Agar/H_2_O	2	13 ± 1.6	18 ± 1.8	0.43 ± 0.02
Agar/H_2_O	4	110 ± 52	270 ± 58	0.37 ± 0.05

It can be noted from Table 2 that the yield stress value increases with a rise in agar concentration. As seen from earlier discussions, at higher concentrations, the supramolecular structures are greatly strengthened and hence the material requires higher stress to sustain its flow. The observed yield stress values conform to other polysaccharide based hydrogels reported in the literature.^129−139^ Additionally, El-hoshoudy and researchers^88,89,131^ mentioned that flow index values, *f* < 1.0 are indicative of pseudoplasticity with shear-thinning behavior. As seen from Table 2, the *f* values reflect this nature of agar dissolved in water. Similar values and behavior are observed for other biopolymer gels.^88,89,131^

**Figure 8:**
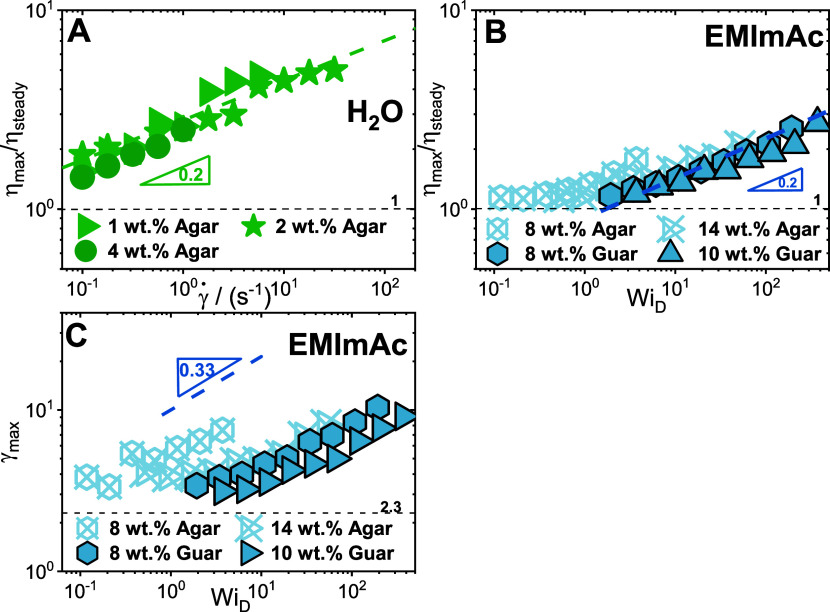
Transient shear response of polysaccharides in different solvent. (A) Ratio of η_max_ and η_steady_ as a function of shear rates for agar in water. The dashed green line represents a power law with a slope of 0.2 as observed for entangled polymer systems. The dashed black line represents the value of 1. (B) Ratio of η_max_ and η_steady_ as a function of *Wi*_D_ (= τ_D_ × γ̇) for polysaccharides in EMImAc. The dashed blue line represents a power law with a slope of 0.2 as observed for entangled polymer systems. The dashed black line represents the value of 1. (C) Strain values at the viscosity overshoot as a function of the *Wi*_D_ (= τ_D_ × γ̇) for polysaccharides in EMImAc. The dashed blue line represents a power law with slope of 0.33 as observed in various polymer systems. The dashed black line indicates the value of 2.3, representative of the Doi–Edwards prediction of tube orientation.

The start-up of shear experiments can be utilized to study the transient shear response of these polysaccharides as shown in Figure 8. First, the fractional overshoot which is the ratio of the peak of transient viscosity (η_max_) values to its steady state (η_steady_) values is studied. The decrease in viscosity after its overshoot in a start-up of shear rate experiment denotes the polymer chain retraction (partial disentanglement or tumbling) and hence the fractional overshoot (η_max_/η_steady_) gives the extent of deformability of the polysaccharides under shear.^140,141^ This fraction is plotted for agar in water as a function of the shear rate in Figure 8A. It is possible to observe that for agar in water the fractional overshoot scales as with the power of 0.2.

In Figure 8B, the fractional overshoot is plotted for the polymers in EMImAc as a function of Weissenberg number (*Wi*_D_ = τ_D_ × γ̇). At lower *Wi*_D_ values (<1), the relative viscosity (η_max_/η_steady_) remains 1 with the polymers depicting no dependence on shear. For *Wi*_D_ > 1, the fractional overshoot scales with a power of 0.2. Similar slopes have been reported for entangled semiflexible linear polymer chains, unentangled H polymer melts, and entangled ring polymers.^142−144^ We have observed that the agar forms higher-ordered structures in water which are disrupted by shear as realized through Cox–Merz failure in Figure 5A. Additionally, we also know that in EMImAc the formation of these supramolecular structures is prevented and the Cox–Merz rule is validated (Figure 5B). Hence, agar chains in water exist as three-dimensional networks formed through helical bundles/supramolecular structures whereas in EMImAc they exist as entangled flexible associating chains. Nevertheless, we can notice that the shear has a very similar effect (identical slopes of 0.2 in Figures 8A, and 8B) on the deformability of the agar irrespective of the solvent media. It is intriguing to observe this similar degree of anisotropy of agar chains in a gel state or liquid state, implying that the effective deformation of the agar chains remains identical irrespective of its solvent.

In Figure 8C, the strain values at the viscosity overshoot (γ_max_) are plotted as a function of *Wi*_D_ for the polysaccharides in EMImAc. The strain value γ_max_ refers to the maximum deformation at the static yielding as opposed to fractional overshoot (η_max_/η_steady_) that corresponds to deformation at steady state, where shear and entropic forces counterbalance.^140^ It is established that at lower *Wi*_D_ values (*Wi*_D_ < 1), shear contributes primarily to tube orientation, and the Doi–Edwards model^145^ predicted that the γ_max_ remains constant and holds a value of 2.3. However, it can be observed in Figure 8C that at *Wi*_D_ values less than 1, the γ_max_ values remain constant at a value of ∼3.5 (>2.3 predicted by Doi–Edwards for tube orientation^145^), most likely reflecting the more complex chain–chain interactions in the polysaccharides, such as intermolecular associations. At faster flows, it is expected that both orientation and chain stretching control the overshoot and hence the γ_max_ should scale up with the *Wi*_D_. Interestingly, at *Wi*_D_ > 1, the γ_max_ increases with *Wi*_D_ and the scaling fairly follows an exponent of 0.33. Comparable slopes have been observed for semiflexible linear polymer chains, weakly associated entangled telechelic star polymers, and entangled ring polymers.^142,143,146^ In conclusion, the above-observed results indicate that irrespective of the kind of polysaccharide and solvent, the shear rate dependence of the maximum deformation prior to yielding masks the structural complexities of natural polysaccharides in quiescent conditions.

An outline of the various rheological functions uncovered in this work through linear and nonlinear shear rheology have been enumerated in Table 3.

**Table 3:** Overview of Scaling Laws in the Rheology of Agar and Guar Gum Dissolved in Water and the Ionic Liquid, 1-Ethyl-3-methylimidazolium Acetate (EMImAc)^a^

polymer	solvent	phase behavior	*G*_N_^0^ ∼ *c^p^*	τ_D_ ∼ *c^q^*	η_sp_ ∼ *c^r^*	η_max_/η_steady_ ∼ γ̇*^s^*/*Wi*_D_*^s^*	γ_max_ ∼ *Wi*_D_*^t^*
agar	water	gel	3			0.2	
guar	water	viscoelastic liquid	3				
agar	EMImAc	viscoelastic liquid	2.3	5.3	7.6	0.2	0.33
guar	EMImAc	viscoelastic liquid	2.3	5.3	7.6	0.2	0.33

a Plateau modulus, *G*_N_^0^; polymer concentration, *c*; terminal relaxation time, τ_D_; specific viscosity, η_sp_; fractional overshoot, η_max_/η_steady_; shear rate, γ̇; terminal Weissenberg number, *Wi*_D_ (= τ_D_ × γ̇); strain value at viscosity overshoot, γ_max_. *p*, *q*, *r*, *s*, and *t* denote the scaling power of the respective rheological functions.

## Conclusions

Two polysaccharides with diverse hydrophilicity were rheologically investigated via linear and nonlinear shear rheology. The study demonstrated how changes in the solvent can significantly influence the dynamics and phase behavior of natural polysaccharides.

In water, agar formed a hydrogel with dynamic moduli values that were independent of frequency, at all tested polymer concentrations. Conversely, guar gum in water behaved as a viscoelastic liquid, but due to its aggregated structure, it exhibited “hyperentanglement” or slow relaxation modes that were clearly observable at very low frequencies. Both polysaccharides in water exhibit power-law dependence of the plateau modulus (*G*_N_^0^) with concentration (*c*) as *G*_N_^0^ ∼ *c*^3^, which can be attributed to the high-order structures formed by the polysaccharides in this solvent.

Interestingly, when dissolved in 1-ethyl-3-methylimidazolium acetate (EMImAc), both polysaccharides formed viscoelastic liquids with significantly lower moduli compared to their counterparts in water. In EMImAc, both polysaccharides obeyed the scaling law *G* ∼ *c*^2.3^, consistent with the theoretical prediction of semiflexible linear polymer chains in good or θ conditions. However, the concentration dependence of both terminal relaxation time (τ_D_) and specific viscosity (η_sp_) revealed that, τ_D_ ∼ c^5.3^ and η_sp_ ∼ c^7.6^, implying that agar and guar gum in EMImAc are associating polymers that adhere the sticky-blob model established by Parisi et al.^86^

The nonlinear response of the polysaccharides was investigated through start-up of shear measurements. Agar in water did not obey the Cox–Merz rule, while guar gum in water only deviated at higher concentrations due to the presence of aggregates. However, in EMImAc, both polysaccharides conformed to the empirical relationship within the investigated concentration and frequency regimes. For agar hydrogels, yield stress values were derived from Herschel-Bulkley fits of the steady-state stress values as a function of shear rate, showing the expected increase in yield stress with higher polysaccharide concentrations. Additionally, the fractional overshoot (η_max_/η_steady_) for agar hydrogel scaled with the shear rate following a power law of 0.2. The deformability of agar and guar gum in EMImAc exhibited a nearly identical scaling relationship with the terminal Weissenberg number (*Wi*_D_). The Doi–Edwards model predicted that the maximum strain at the static yielding (γ_max_) remains constant, with a value of 2.3 at terminal Weissenberg numbers *Wi*_D_ < 1, due to tube orientation. However, the γ_max_ values of both polysaccharides in EMImAc remain constant at around 3.5, likely reflecting the more complex chain–chain interactions in the polysaccharide chains, such as intermolecular associations. For *Wi*_D_ > 1, γ_max_ for agar and guar gum shown a scaling of 0.33 with *Wi*_D_, similar to that reported for synthetic polymeric systems with various molecular architectures. This suggests that under fast flows, the shear rate dependence of the maximum deformation prior to yielding masks the structural complexities of natural polysaccharides in quiescent conditions.

In addition to the above established scaling relations, further investigation into these biopolymer systems could yield deeper insights. Specifically, X-ray scattering techniques could be employed to characterize the presence or absence of supramolecular structures in both water and EMImAc. Scattering studies could provide a more detailed understanding of the ‘hyperentanglements’ exhibited by guar gum in water. Furthermore, spectroscopy techniques could be utilized to distinguish the changes in activation energy of these polymers when dissolved in EMImAc as opposed to water. Given that water and EMImAc are miscible, a binary solvent system can be prepared by varying their composition, with the subsequent addition of biopolymers to fine-tune the rheological properties of these materials. This approach offers significant opportunities to create materials that combine the advantageous properties of both solvents.

In conclusion, our findings provide valuable insights into the rheological behavior of natural polysaccharides and underscore the role of the solvent selection in tailoring their rheological properties for various applications.

## Supporting Information Available

Polysaccharide molecular structure; linear viscoelasticity (additional frequency and time sweeps); start-up of shear measurements (stress growth coefficient vs time) (PDF)
