# Supplementary material for: Scaling Laws in Polysaccharide Rheology: Comparative Analysis of Water and Ionic Liquid Systems
Source: Biomacromolecules. 2024 Sep 16;25(10):6883–98. doi: 10.1021/acs.biomac.4c01125 (PMC11480991; doi:10.1021/acs.biomac.4c01125)
Supplement: Supplementary file 1 — bm4c01125_si_001.pdf [file bm4c01125_si_001.pdf]

Supporting Information

Scaling Laws in Polysaccharide Rheology: Comparative Analysis of Water and Ionic Liquid Systems

Roshan Akdar Mohamed Yunus[1], and Daniele Parisi[1,*]

[1]*Department of Chemical Engineering, Engineering and Technology institute Groningen, University of Groningen, Nijenborgh 3, 9747 AG Groningen, The Netherlands*

*Corresponding author: d.parisi@rug.nl

Polysaccharide Structure

The two-polysaccharide employed in this work are agar and guar gum. Agar is composed of two different fractions – agarose and agaropectin. Agarose is the primary component of agar and responsible for gelling properties of agar. Guar gum is a copolymer belonging to the family of galactomannans containing monomeric units of mannose and galactose. The structure of these two polysaccharides is shown in Figure S1.

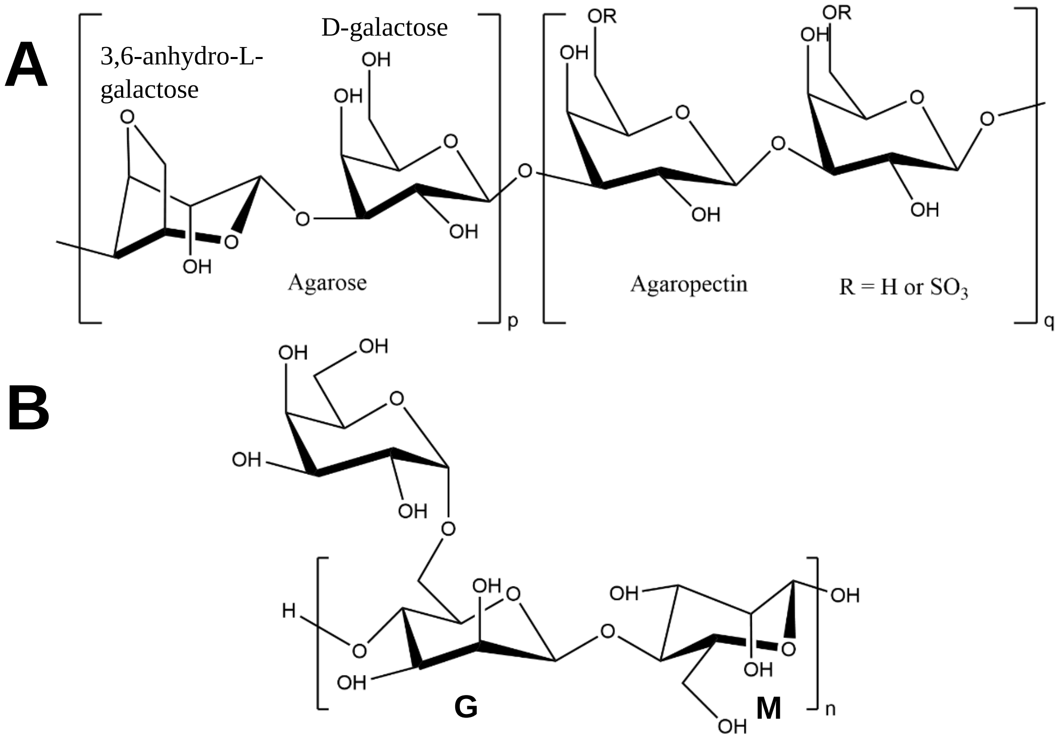

Figure S1. Structure of polysaccharides. (A) Agar comprising of agarose and agaropectin with p and q degree of polymerization, respectively. In agaropectin, the R group can have either H or SO_3 groups. (B) Guar gum comprising of mannose (M) and galactose substituted mannose (G) with a degree of polymerization of n.

Linear viscoelasticity

Figure S2 depicts the rheological spectrum in terms of storage G' and loss G" modulus as a function of the oscillation frequency ω, for guar/water solution at 4 wt.% polymer content. The crossover at the lower frequency and consequently the second elastic plateau is indicative of "hyperentanglements" that account for relaxation mechanisms that exceed the disentanglement of the "reptated" chain.

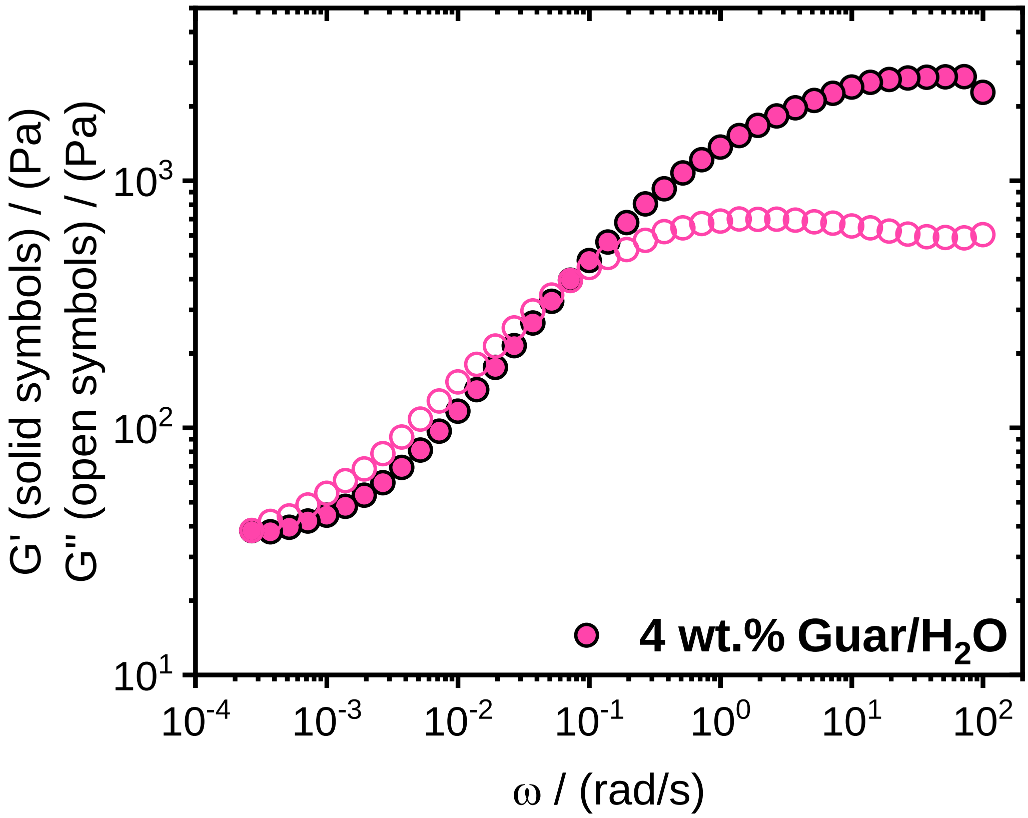

Figure S2. Storage G′ (closed symbols) and loss G″ (open symbols) modulus as a function of oscillation frequency ω for aqueous solution of guar gum at 4 wt.% of polymer mass fraction. The experiment was performed over an extended range of frequency and at 35 °C.

Figure S3 shows the rheological spectrum in terms of storage G' and loss G" modulus as a function of the oscillation frequency ω, for guar/EMImAc solution at 5 wt.% polymer content. No low-frequency crossover can be observed.

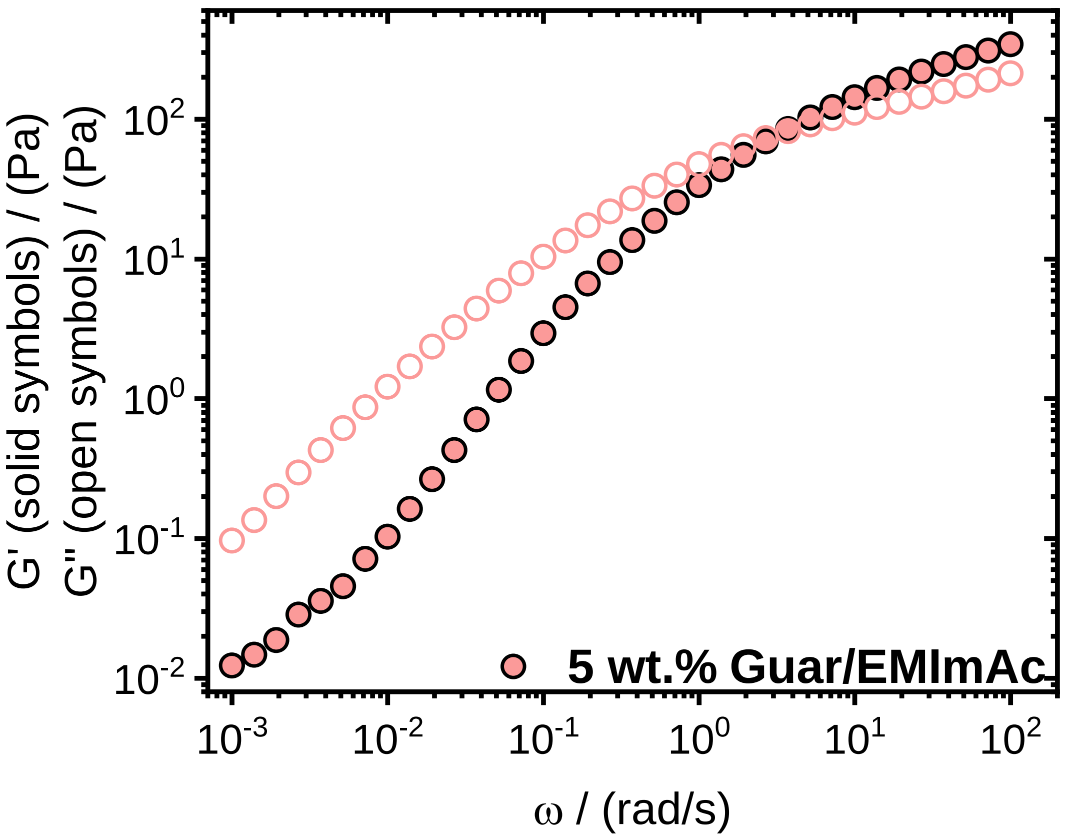

Figure S3. Storage G′ (closed symbols) and loss G″ (open symbols) modulus as a function of oscillation frequency ω for guar gum dissolved in EMImAc at 5 wt.% of polymer mass fraction. The experiment was performed over an extended range of frequency and at 35 °C.

Nonlinear viscoelasticity

The non-linearity of agar and guar gum in both solvents – water and 1-ethyl-3-methylimidazolium acetate (EMImAc) were investigated through start-up of shear experiments, reported in Figures S4-S8.

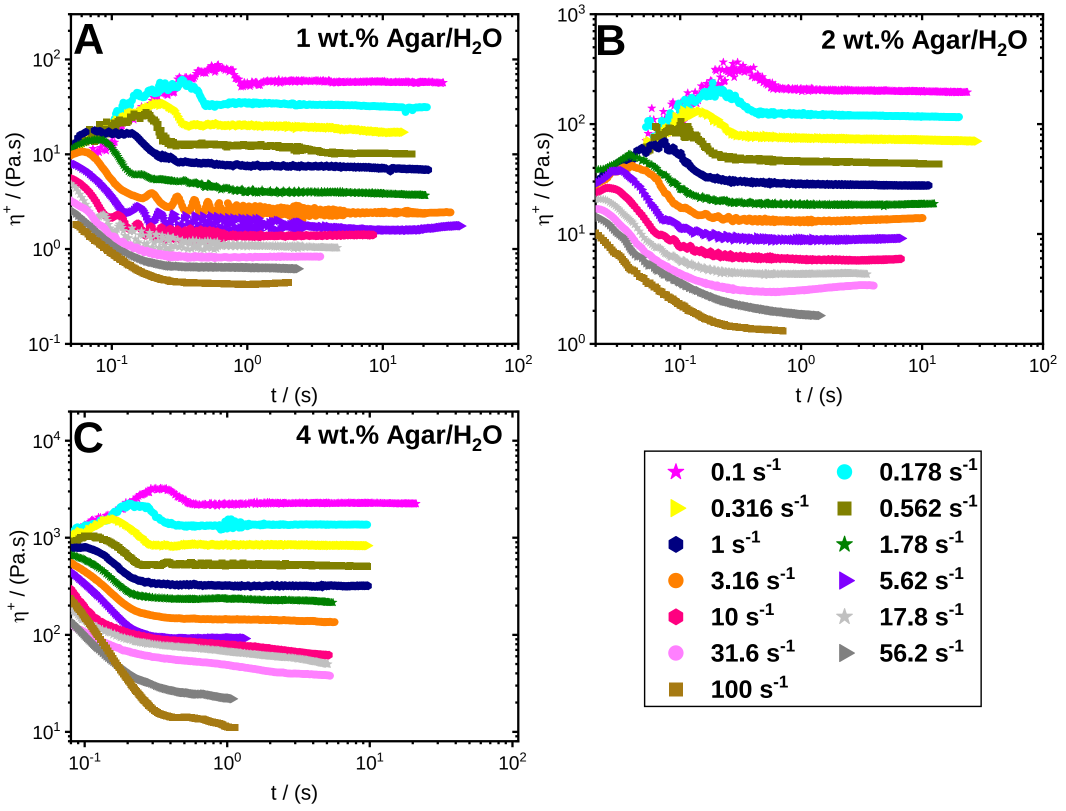

Figure S4. Start-up of shear rate in terms of the stress growth coefficient as a function of time at various shear rates (reported in the legend) for agar hydrogels at (A) 1 wt.%, (B) 2 wt.%, and (C) 4 wt.%. Experiments were performed at 35 °C.

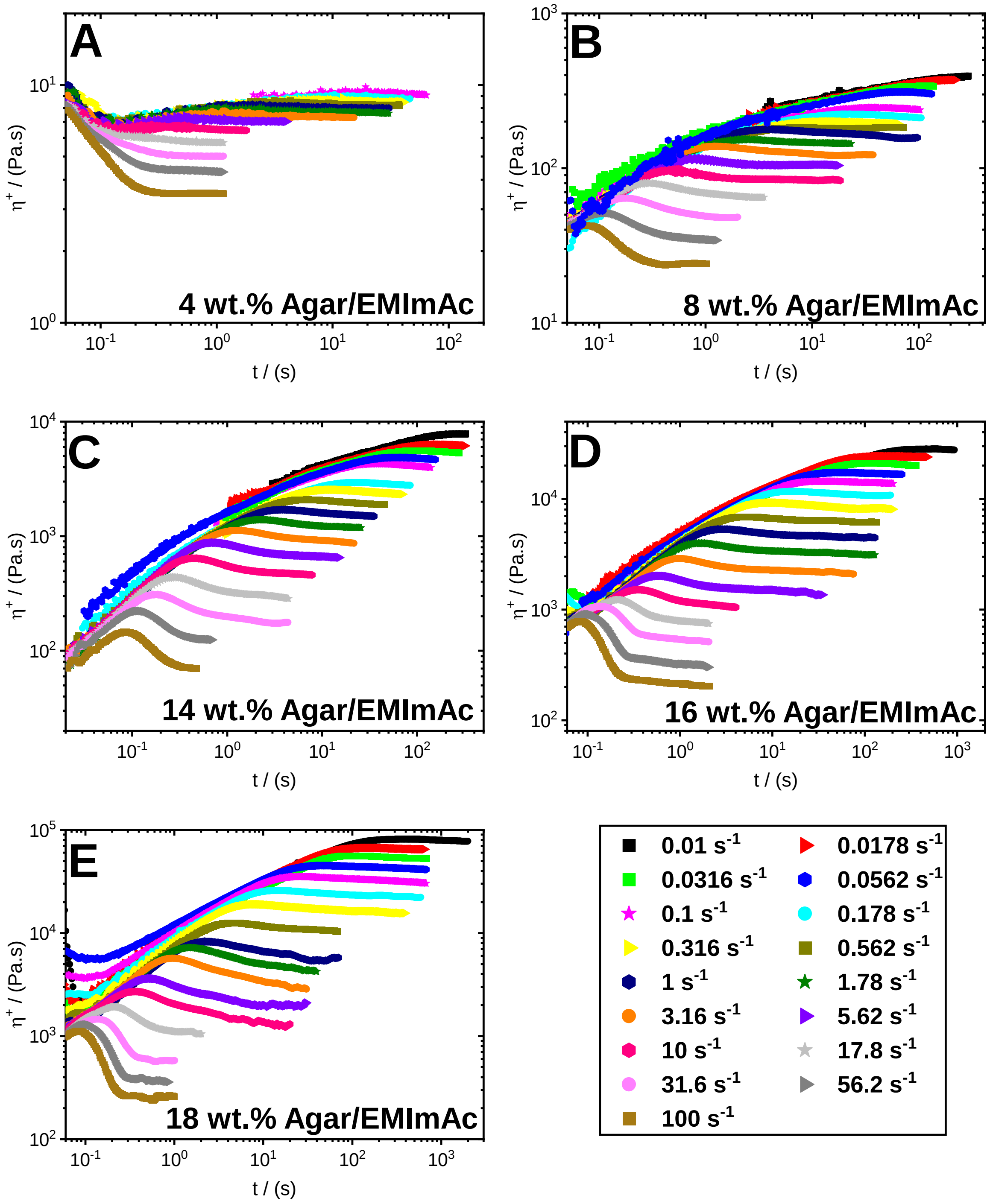

Figure S5. Start-up of shear rate in terms of the stress growth coefficient as a function of time at various shear rates (reported in the legend) for agar dissolved in EMImAc at (A) 4 wt.%, (B) 8 wt.%, (C) 14 wt.%, (D) 16 wt.%, and (E) 18 wt.%. Experiments were performed at 35 °C.

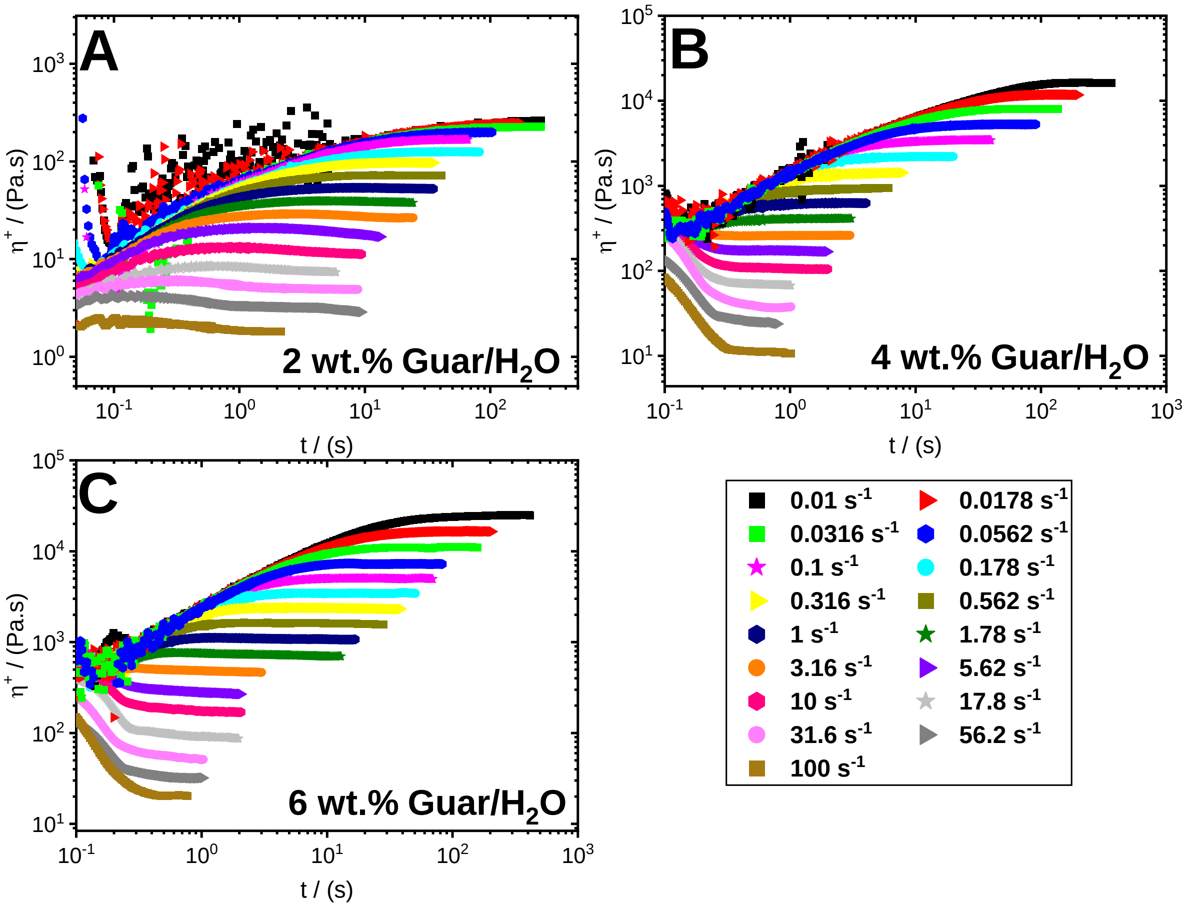

Figure S6. Start-up of shear rate in terms of the stress growth coefficient as a function of time at various shear rates (reported in the legend) for guar dissolved in water at (A) 2 wt.%, (B) 4 wt.%, and (C) 6 wt.%. Experiments were performed at 35 °C.

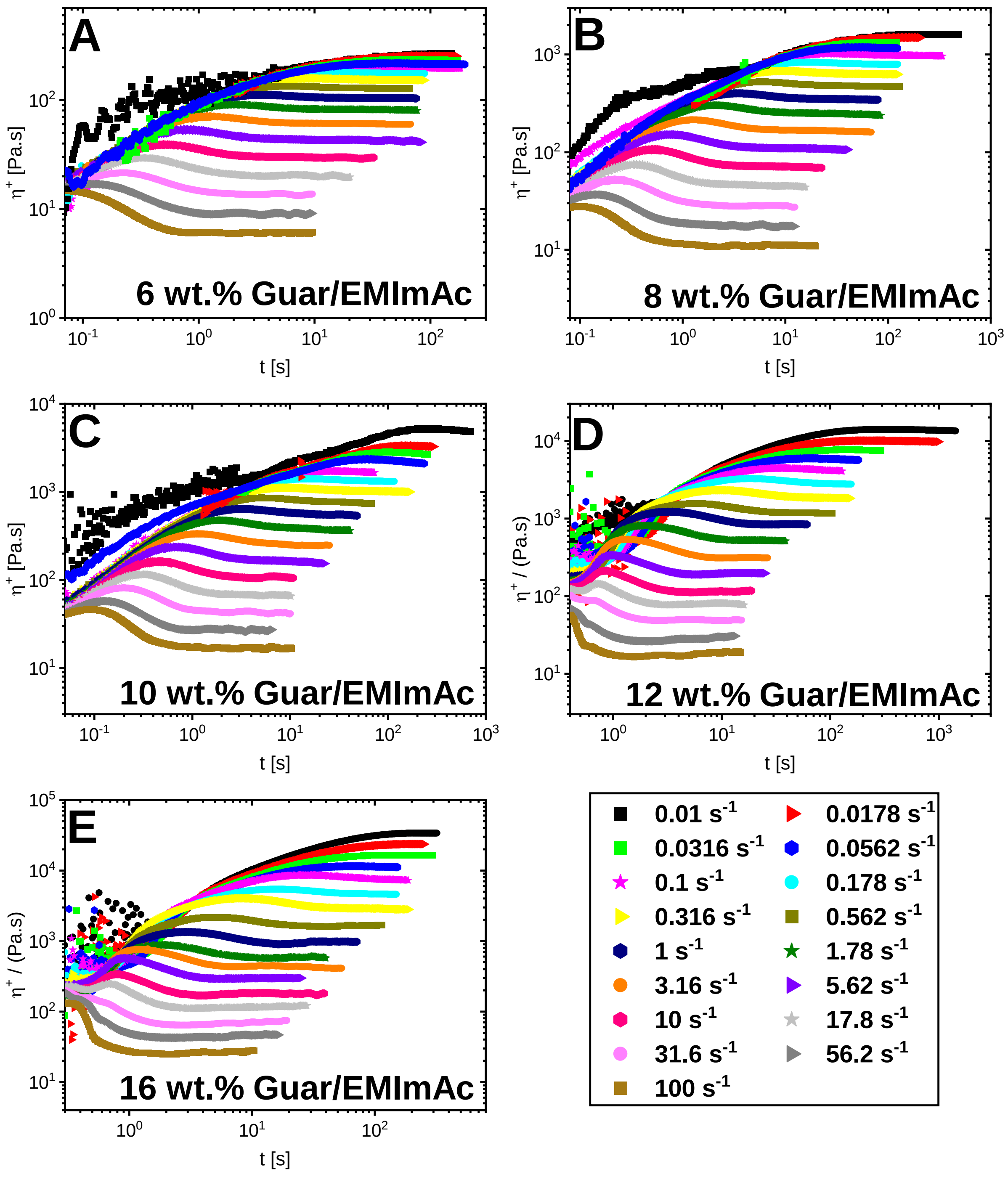

Figure S7. Start-up of shear rate in terms of the stress growth coefficient as a function of time at various shear rates (reported in the legend) for guar gum dissolved in EMImAc at (A) 6 wt.%, (B) 8 wt.%, (C) 10 wt.%, (D) 12 wt.%, and (E) 16 wt.%. Experiments were performed at 35 °C.

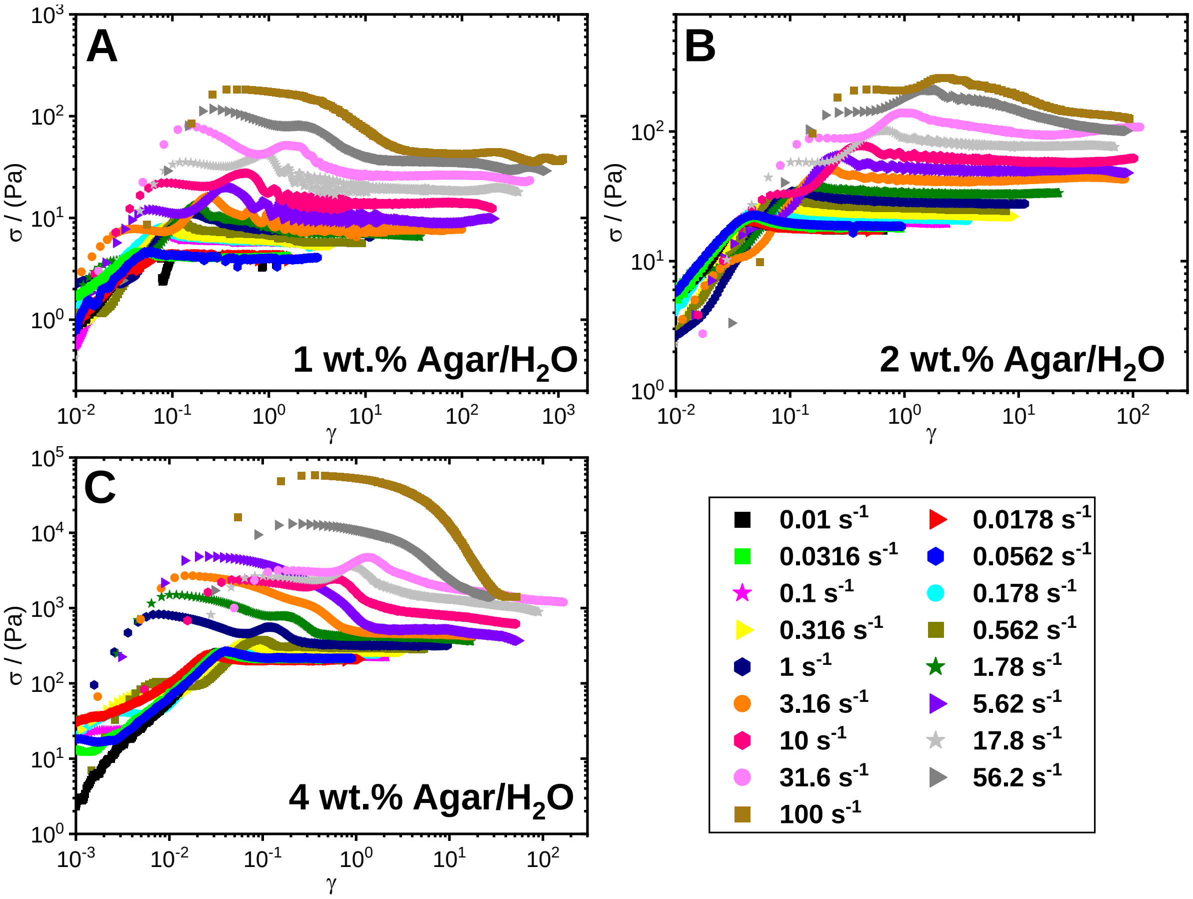

Figure S8. Start-up of shear rate in terms of the stress response as a function of strain at various shear rates (reported in the legend) for agar dissolved in water at (A) 1 wt.%, (B) 2 wt.%, and (C) 4 wt.%. Experiments were performed at 35 °C.

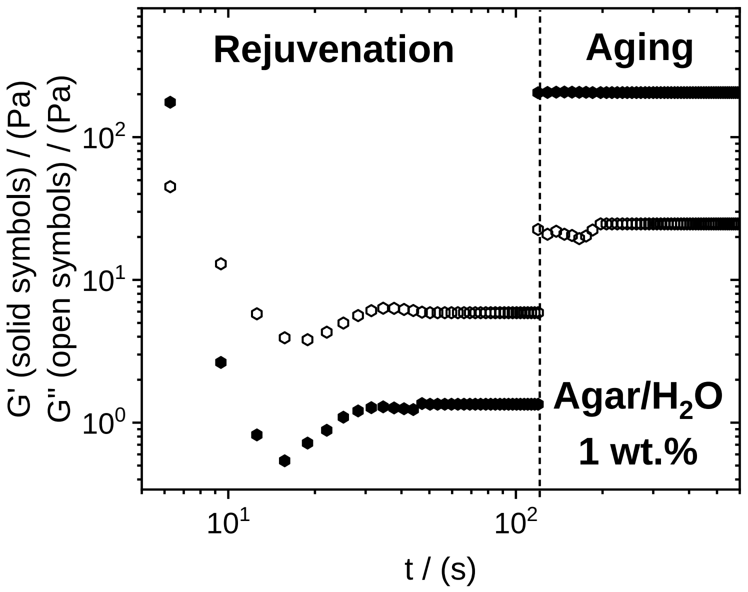

Figure S9. Dynamic time sweep (DtS) at 1 rad/s for 1 wt.% of agar dissolved in water. It depicts the structural breakdown of the sample at 100% strain and the aging of the sample with strain within the linear viscoelastic regime.

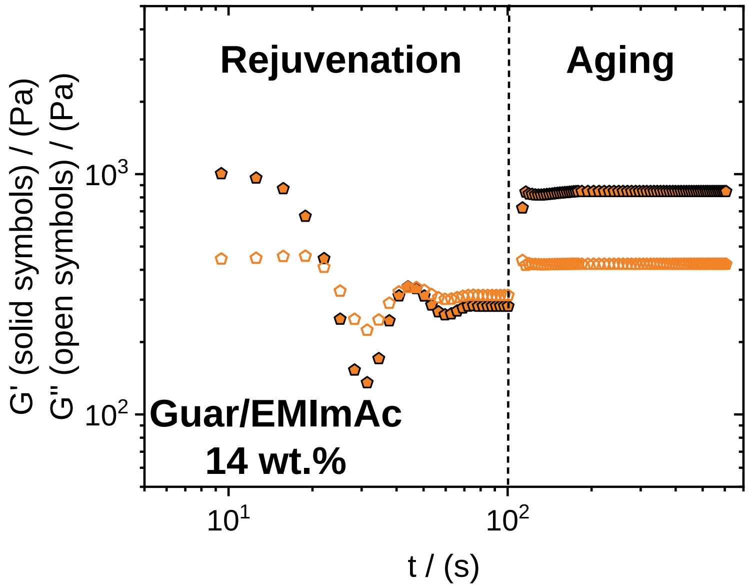

Figure S10. Dynamic time sweep (DtS) at 1 rad/s for 14 wt.% of guar gum dissolved in EMImAc. It depicts the structural breakdown of the sample at 300% strain and the aging of the sample with strain within the linear viscoelastic regime.
